# Long-Term Outcome after Early Mammalian Target of Rapamycin Inhibitor-Based Immunosuppression in Kidney Transplant Recipients

**DOI:** 10.3390/jcm13154305

**Published:** 2024-07-23

**Authors:** Lutz Liefeldt, Johannes Waiser, Friederike Bachmann, Klemens Budde, Frank Friedersdorff, Fabian Halleck, Nils Lachmann, Robert Peters, Birgit Rudolph, Sinem Ünlü, Kaiyin Wu, Petra Glander

**Affiliations:** 1 Department of Nephrology and Internal Intensive Care Medicine, Charité—Universitätsmedizin Berlin, Campus Charité Mitte, Charitéplatz 1, 10117 Berlin, Germany; johannes.waiser@charite.de (J.W.); friederike.bachmann@charite.de (F.B.); klemens.budde@charite.de (K.B.); fabian.halleck@charite.de (F.H.); kaiyin.wu@charite.de (K.W.); petra.glander@charite.de (P.G.); 2 Department of Urology, Charité—Universitätsmedizin Berlin, 10117 Berlin, Germany; frank.friedersdorff@charite.de (F.F.); robert.peters@charite.de (R.P.); 3 Centre for Tumor Medicine, H&I Laboratory, Charité—Universitätsmedizin Berlin, 13353 Berlin, Germany; nils.lachmann@charite.de (N.L.); sinem.uenlue@charite.de (S.Ü.); 4 Department of Pathology, Charité—Universitätsmedizin Berlin, 10117 Berlin, Germany; birgit.rudolph@charite.de

**Keywords:** early immunosuppression, kidney transplantation, mammalian target of rapamycin inhibitors, calcineurin inhibitors, long-term outcome

## Abstract

**Background:** The use of mammalian target of rapamycin inhibitors (mTORis) in kidney transplantation increases the risk of donor-specific human leukocyte antigen (HLA) antibody formation and rejection. Here, we investigated the long-term consequences of early mTORi treatment compared to calcineurin inhibitor (CNI) treatment. **Methods:** In this retrospective single-center analysis, key outcome parameters were compared between patients participating in randomized controlled immunosuppression trials between 1998 and 2011, with complete follow-up until 2018. The outcomes of eligible patients on a CNI-based regimen (n = 384) were compared with those of patients randomized to a CNI-free mTORi-based regimen (n = 81) and 76 patients randomized to a combination of CNI and mTORi treatments. All data were analyzed according to the intention-to-treat (ITT) principle. **Results:** Deviation from randomized immunosuppression for clinical reasons occurred significantly more often and much earlier in both mTORi-containing regimens than in the CNI treatment. Overall patient survival, graft survival, and death-censored graft survival did not differ between the treatment groups. Donor-specific HLA antibody formation and BPARs were significantly more common in both mTORi regimens than in the CNI-based immunosuppression. **Conclusions:** The tolerability and efficacy of the mTORi treatment in kidney graft recipients are inferior to those of CNI-based immunosuppression, while the long-term patient and graft survival rates were similar.

## 1. Introduction

The use of the calcineurin inhibitors (CNIs) cyclosporine or tacrolimus in combination with anti-IL-2 receptor antibodies (e.g., basiliximab) and mycophenolic acid (MPA; e.g., mycophenolate mofetil (MMF) or enteric-coated mycophenolate sodium (EC-MPS)), with or without steroids, has led to major advances in renal transplantation [1] with excellent short-term outcomes, and they are recommended by current guidelines for immunologically low-risk patients [2]. However, CNIs are nephrotoxic and are a frequent secondary cause of graft loss [3]. The development of mammalian target of rapamycin inhibitors (mTORis) has led to the availability of less nephrotoxic immunosuppressive drug combinations. Sirolimus (rapamycin) is a macrocyclic triene antibiotic produced by the fermentation of Streptomyces hygroscopicus. Sirolimus was discovered in a soil sample collected from Rapa Nui, also known as Easter Island [4]. Sirolimus has been approved as an initial immunosuppressive treatment after kidney transplantation in combination with cyclosporine and corticosteroids [5]. After 2–3 months, the cyclosporine dose should be tapered and eventually withdrawn. Thereafter, the regimen should consist of sirolimus and corticosteroids [6].

Everolimus in combination with cyclosporine and corticosteroids has been approved as an initial immunosuppressive treatment after kidney and heart transplantations. Furthermore, it has been approved for use in liver allograft recipients in combination with tacrolimus and corticosteroids. Notably, the withdrawal of CNIs is not recommended in everolimus-containing regimens after solid organ transplantation. To avoid the renal toxicity of CNIs in combination with everolimus, it has been recommended to reduce cyclosporine or tacrolimus early after transplantation [7].

Twenty years after the approval of sirolimus (Europe, 2001; US, 2003) and its derivative everolimus (Europe, 2004; US, 2010), the mTORi principle is rarely used and is still seeking its place in clinical kidney transplantation. Side-effects are frequent, and KDIGO found no clear advantage in mTORis in comparison with other antiproliferative agents or as a replacement for or combination with CNIs [2]. Our center has participated in many clinical trials of mTORis, and we observed an increased risk of de novo donor-specific HLA antibody (dnDSA) formation and antibody-mediated rejection in patients treated with everolimus compared to those treated with cyclosporine [8]. More recently, studies from the US and Australia, as well as a meta-analysis, have described increased mortality among kidney transplant recipients treated with mTORi-based immunosuppression [9,10,11,12]. These and other recent investigations on the use of everolimus in combination with reduced CNI exposure [13,14,15] or standard-dose CNIs [16,17] in renal transplantation led us to re-evaluate and extend our previous analyses. We aimed to investigate the long-term consequences of early mTORi use (either in combination with CNIs or in a CNI-free regimen) compared with standard CNI-based immunosuppression.

## 2. Methods

In this retrospective single-center analysis, we studied the outcomes of patients who participated in a number of clinical trials at our center and who were primarily randomized to CNIs, a CNI-free mTORi-based regimen, or a combination of CNIs and mTORis. Thus, the analysis benefits from the stringent exclusion criteria, the randomized use of immunosuppressive agents, and complete long-term follow-up in our database.

### 2.1. Patients

To identify as many patients eligible for this study as possible, all adult recipients of a kidney allograft transplanted between 10 September 1998 and 8 February 2011 were screened. In 1998, the first patient was randomized to an mTORi in a clinical trial. By 8 February 2011, the last transplantation of a patient randomized to receive mTORi-based immunosuppression was performed. To exclude patients with early intolerability, we included only those with at least 4 weeks of randomized treatment with CNIs (intention-to-treat (ITT) group 1), mTORis (ITT group 2), or a combined treatment with CNIs and mTORis (ITT group 3). The prospective randomized controlled trials comparing different immunosuppressant regimens are summarized in Table 1 [18,19,20,21,22,23,24,25,26,27,28,29,30,31,32,33,34,35,36,37,38,39,40,41,42]. The decision for transplantation was based on a negative CDC crossmatch and the absence of DSAs at the time of transplantation. The dosing of CNIs and mTORis was based on trough levels and in accordance with study protocols. Initially, standard doses of mycophenolic acid were administered, and later individualized on the basis of side-effects and tolerability. Most trials allowed for a steroid treatment according to center policy. Our center policy is to administer steroids for at least 12 months. Thereafter, the withdrawal of steroids is based on individual decision making, considering the immunological risk (namely, previous rejections), side-effects, underlying renal disease, and patient preferences.

### 2.2. HLA Antibody Screening

HLA antibody screening was performed on the discretion of the renal physician responsible for the follow-up and at the time of indication of graft biopsies. In addition, serum samples were prospectively collected at least once a year for the HLA antibody screening starting in 2006. All of the serum samples were qualitatively screened for HLA antibodies using the Luminex-based bead assay LABScreen Mixed (One Lambda; Canoga Park, CA, USA). Donor specificity of the HLA antibodies was determined using LABScreen Single Antigen Beads (One Lambda). All of the tests were performed in accordance with the manufacturer’s guidelines.

### 2.3. Graft Biopsies

All of the graft biopsies were performed for clinical indications, such as impaired kidney function and/or proteinuria. Renal allograft pathology was performed by two experienced nephropathologists (B.R. and K.W.) according to the Banff classification. All of the histological findings were re-categorized in 2010 according to the Banff 09 classification [43], and later to the Banff 2017 classification [44].

### 2.4. Follow-Up

All of the patients underwent regular follow-up at our outpatient clinic. All of the clinical data were retrieved from the web-based electronic patient record system TBase [45]. The end of the observation period was June 30th, 2018.

The Institutional Review Board of Charité—Universitätsmedizin Berlin approved the retrospective analysis of patient data, with a waiver of informed consent (EA1/048/14).

### 2.5. Statistics

We used IBM SPSS Statistics for Windows version 27.0. (IBM Corp., Armonk, NY, USA) for all of the statistical analyses. Descriptive data were summarized as medians with ranges. Categorical data were presented as proportions.

The endpoints were death, graft loss, de novo DSA formation, and first biopsy-proven acute rejection (BPAR) episodes after the first month. Events over time (Kaplan–Meier) were analyzed using the log-rank test.

Cox regression analyses were used to identify prognostic factors that contributed to the endpoint variability. Therefore, univariate analyses with a number of factors that potentially affect the outcomes were performed: recipient age (≥65 vs. <65 years), recipient sex (female vs. male), number of KTXs (repeated vs. first), waiting time (<60 vs. ≥60 months), renal disease (GN vs. other), PRAs pretransplant (positive vs. negative), donor age (≥65 vs. <65), donor sex (female vs. male), donor type (living vs. deceased), number of mismatches (4–6 vs. 0–3), KTX function (DGF vs. primary function), AR within the first month (yes vs. no), ITT group (mTORis, CNIs + mTORis vs. CNIs), steroid stop (yes vs. no), and KTX date (after March 31, 2005 vs. before March 31, 2005). Variables with a *p* < 0.20 in the univariate analysis were included in a multivariate Cox proportional hazard model, and a stepwise backward elimination strategy (*p* > 0.05) was applied to identify the risk factors for overall patient survival, death-censored graft loss, overall graft loss, dnDSA formation, and biopsy-proven acute rejection after the first month.

A probability of less than 0.05 was considered as statistically significant.

## 3. Results

### 3.1. Patient Population

**Figure 1:**
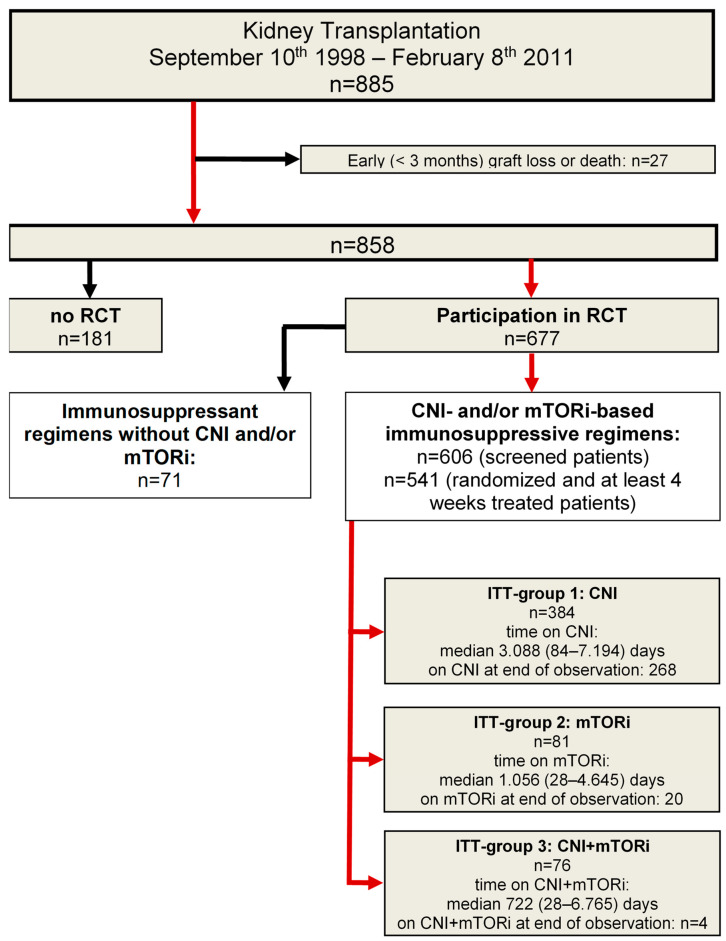
Description of steps towards the definition of a study cohort for the comparison of different immunosuppressant regimens early after kidney transplantation.

Figure 1 illustrates the steps used to define the study cohort. Only patients with at least four weeks of randomized treatment with CNIs (ITT group 1, n = 384), mTORis (ITT group 2, n = 81), or simultaneous treatment with CNIs and mTORis (ITT group 3, n = 76) were included in the study. The baseline characteristics of the intention-to-treat groups are presented in Table 2.

A total of 268 of 384 (69.84%) patients who were randomized to receive a CNI-based regimen remained on CNI maintenance treatment until graft loss, death with a functioning graft, or the end of observation. Of the CNI sub-cohort, 17 patients were switched to mTORis during the first year, and 90 were switched later (after 12 months) on the discretion of their renal physicians. The reasons for the mTORi changeover included neoplasia (n = 23), CNI nephrotoxicity (n = 61), viral infections (n = 6), or other reasons, including participation in other clinical trials (n = 17).

Of the 81 patients who were randomized to receive an mTORi-based regimen, only 20 (24.7%) remained on mTORis until graft loss, death, or the end of observation. In total, 59 patients were switched to CNIs for clinical reasons (mTORi-specific toxicity, n = 22; biopsy-proven acute rejections (BPARs), n = 15; detection of dnDSA formation, n = 12; increased risk of dnDSA formation, n = 10). Two patients were converted to belatacept because of their participation in a clinical trial.

Of the 76 patients who were randomized to mTORis plus CNIs, only 4 (5.3%) remained on this combination until graft loss, death, or the end of observation. Twenty-three patients were switched to other regimens because of CNI toxicity. An mTORi-specific toxicity was mentioned in the records of 14 patients. Ten patients left the protocol after a BPAR diagnosis. Five patients were switched to prevent ABMR after dnDSA formation was detected. Combinations of the mentioned reasons for changeover or other reasons led to the end of the combined treatment in 20 patients.

**Figure 2:**
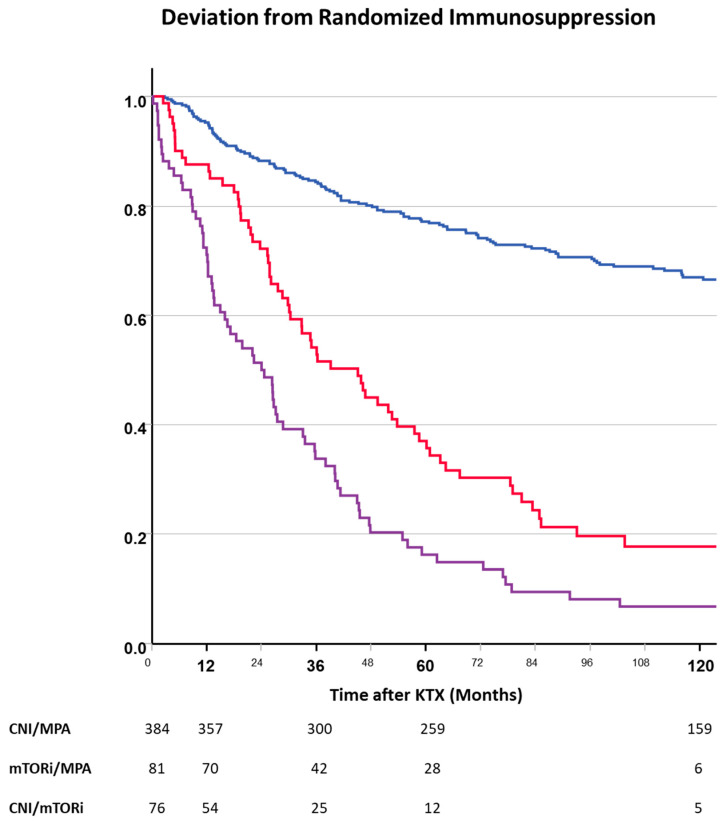
Deviation from randomized immunosuppression after transplantation according to the ITT group (blue—CNI, red—mTORi, purple—CNI + mTORi). Patients of both mTORi groups were more often switched to other immunosuppressive regimens than CNI patients (log-rank: *p* < 0.001 each). Patients on the combination of mTORis plus CNIs were more likely to be changed than patients on mTORis plus MPA (log-rank: *p* = 0.001).

Deviations from randomized immunosuppression were significantly more common and occurred earlier in the mTORi groups than in the CNI-based treatment group (Figure 2).

### 3.2. Patient Survival

**Figure 3:**
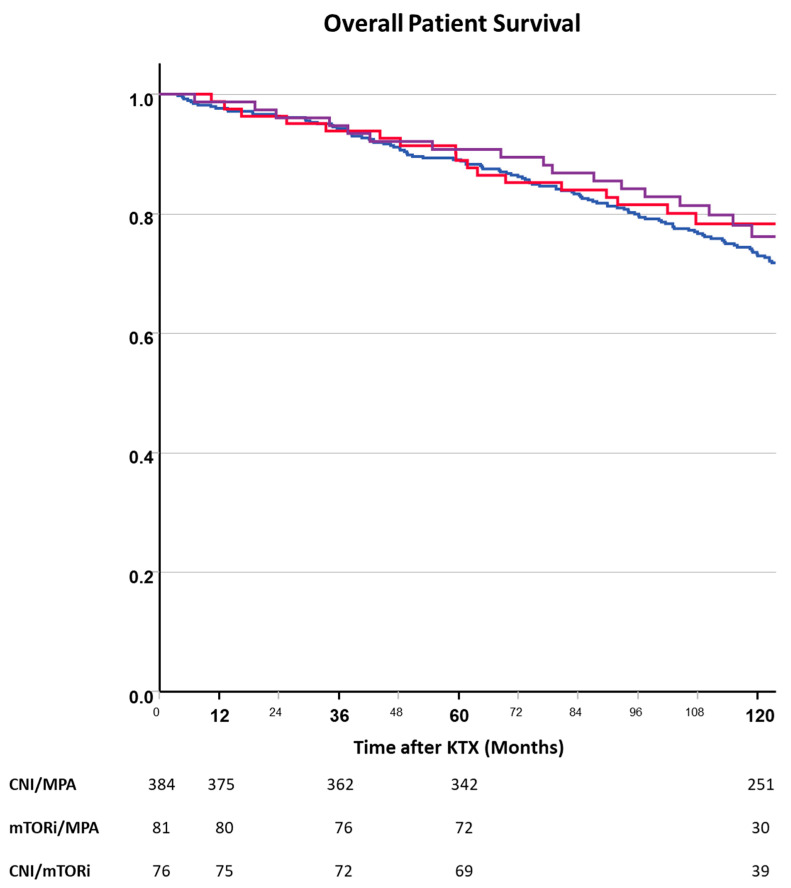
Overall patient survival after kidney transplantation depending on randomized immunosuppressive regimen (blue—CNI, red—mTORi, purple—CNI + mTORi). Patient survival did not differ between the treatment groups.

The risk of death in our cohort was not dependent on early immunosuppression (Figure 3). Sixty months after transplantation, the overall survival rates were 88.9%, 90.8%, and 89.1% in patients treated with mTORis, a combination, and CNI-treated patients, respectively.

Recipient age, underlying renal disease, donor sex, donor type, and decision to withdraw steroids were significant confounders in the multivariate model of patient survival, whereas early immunosuppression was not (Table 3).

### 3.3. Graft Loss

**Figure 4:**
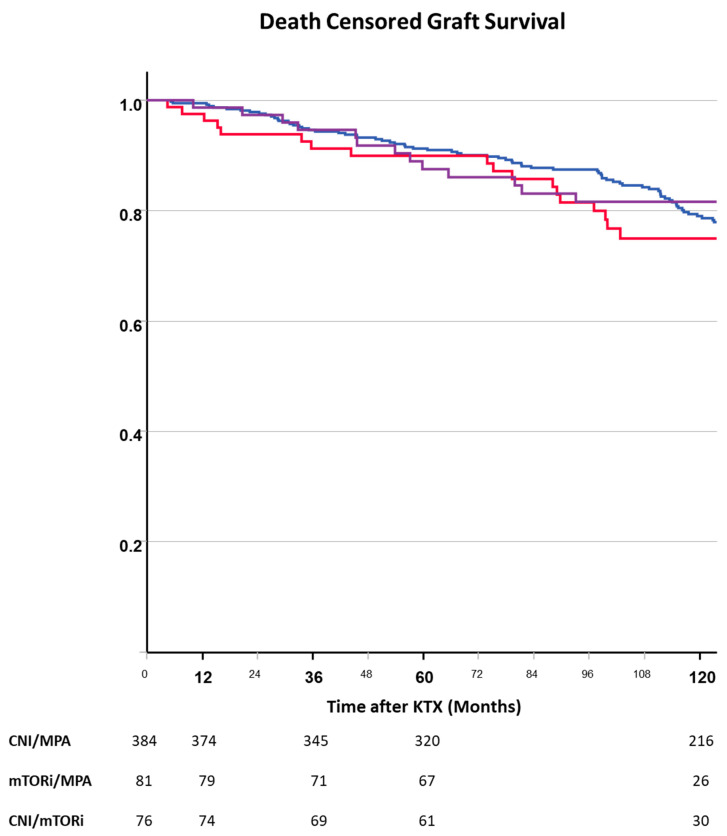
Death-censored graft loss after kidney transplantation depending on randomized immunosuppressive regimen (blue—CNI, red—mTORi, purple—CNI + mTORi). Graft loss did not differ between the randomized immunosuppressive regimens.

The risk of death-censored graft loss was not dependent on early immunosuppression (Figure 4). Recipient age, number of KTXs, and occurrence of DGF increased the risk of death-censored graft loss in the multivariate model, whereas neither type of randomized early immunosuppression did (Table 3). The decision to discontinue steroids was associated with a decreased risk of death-censored graft loss.

**Figure 5:**
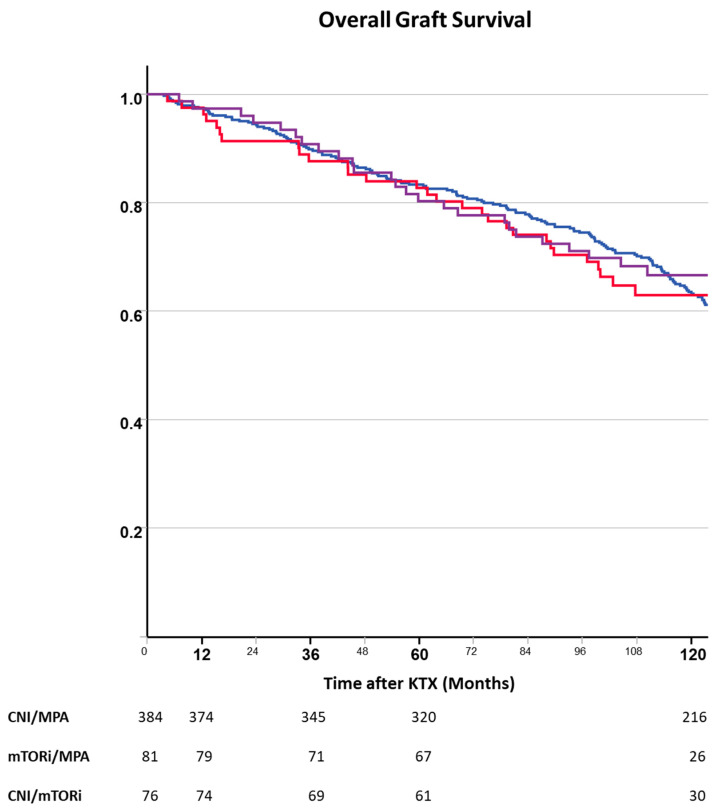
Overall graft loss after kidney transplantation depending on randomized immunosuppressive regimen (blue—CNI, red—mTORi, purple—CNI + mTORi). Overall graft loss did not differ between the randomized immunosuppressive regimens.

Analysis of the overall graft survival did not show significant differences between the ITT groups (Figure 5). Recipient age, donor type, DGF, steroid withdrawal, renal disease, and year of transplantation (era) were significantly associated with overall graft survival (Table 3).

### 3.4. BPAR

**Figure 6:**
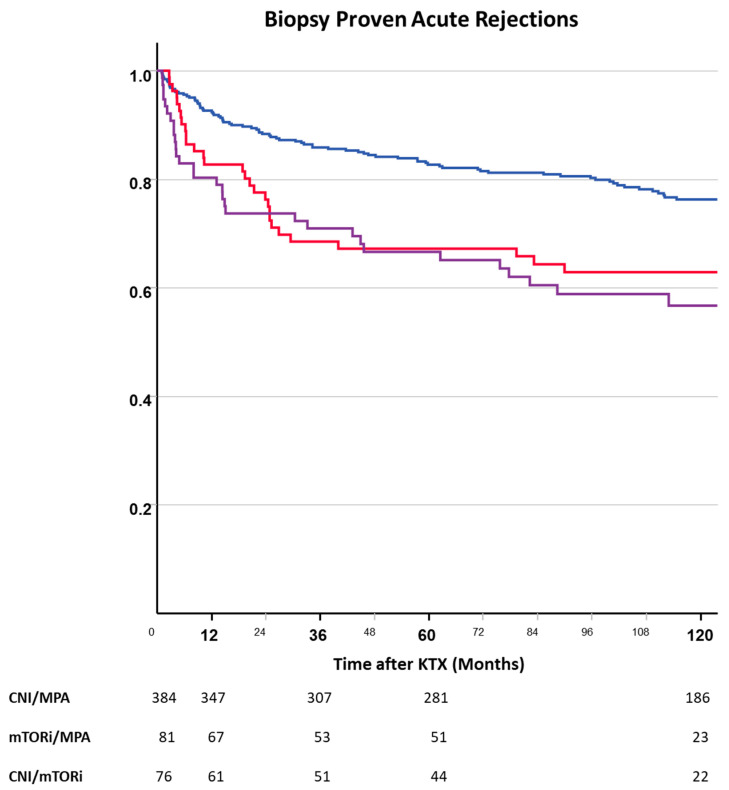
Biopsy-proven acute rejections >1 month after kidney transplantation depending on randomized immunosuppressive regimen (blue—CNI, red—mTORi, purple—CNI + mTORi). mTORi use was significantly more often associated with BPARs (log-rank: mTORi vs. CNI—*p* = 0.004; mTORi + CNI vs. CNI—*p* < 0.001).

Biopsy-proven acute rejection in indication biopsies ≥1 month after transplantation was significantly more common in mTORi-treated patients than in those treated with CNI-based immunosuppression (Figure 6). Sixty months after transplantation, the cumulative rates of the first BPAR more than 1 month after transplantation were 33.8%, 33.4%, and 17.3% in CNI-free mTORi-treated, mTORi + CNI, and CNI-treated patients, respectively.

Risk factors for BPAR in the multivariate analysis included a higher number of mismatches, the occurrence of DGF, and randomized treatment with mTORis. Remarkably, the decision to withdraw steroids from the immunosuppressive maintenance regimen was not associated with increased rejection rates after month 1 (Table 3).

### 3.5. dnDSA Formation

**Figure 7:**
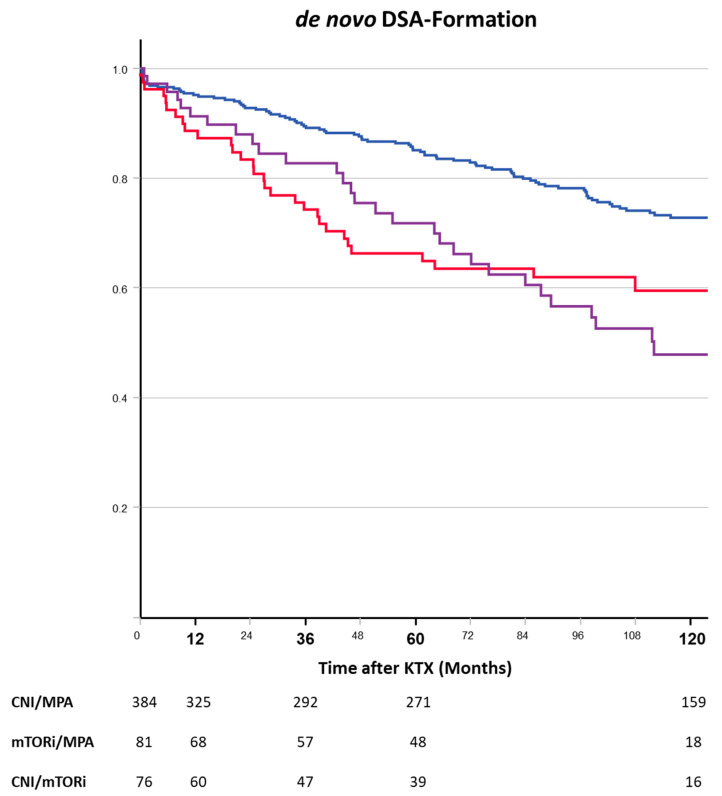
De novo DSA formation after kidney transplantation depending on the randomized immunosuppressive regimen (blue—CNI, red—mTORi, purple—CNI + mTORi). mTORi use was significantly more often associated with dnDSA formation in the ITT analyses (log-rank: mTORi vs. CNI—*p* = 0.006; mTORi + CNI vs. CNI—*p* = 0.001).

De novo DSA formation was significantly more common throughout the observation period in both mTORi-containing regimens than in the CNI group (Figure 7). Sixty months after transplantation, dnDSAs were detected in 35.1%, 30.4%, and 15.6% of CNI-free mTORi-treated, CNI + mTORi, and CNI-treated patients, respectively.

Table 3 summarizes the contributions of different variables to the formation of *de novo* DSAs in the multivariate model. In the final model, the randomized use of mTORis was associated with an increased risk of dnDSA formation, such as pretransplant PRAs, the number of mismatches, and the occurrence of DGF. Remarkably, steroid withdrawal from immunosuppression was associated with less dnDSA formation in the multivariate model.

**Figure 8:**
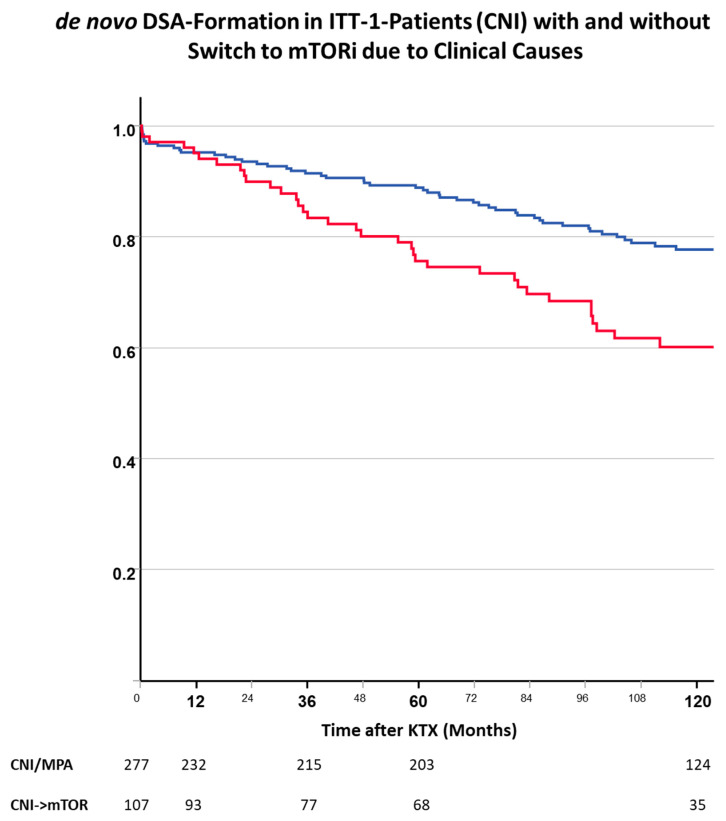
De novo DSA formation after kidney transplantation in patients from ITT group 1 with maintenance CNIs or conversion to a CNI-free, mTORi-based immunosuppression for clinical reasons (blue—ever CNI, red—mTORi-conversion). Maintenance of a CNI-based immunosuppression in comparison with a switch to mTORi (CNI-free) combinations protected from dnDSA formation (log-rank: *p* = 0.002).

In secondary confirmatory analyses, we studied whether clinically initiated conversion from CNIs to mTORis was associated with a higher frequency of dnDSAs. The switch from CNIs (ITT group 1) to mTORis for clinical reasons was also associated with an increased risk of dnDSA formation, similar to the ITT analysis (Figure 8). Patients who continued CNIs over time had the lowest risk of dnDSAs (after 60 months: 11.2% with unchanged CNI treatment vs. 24.4% after conversion to mTORis).

## 4. Discussion

In general, long-term data from randomized trials are sparse. The long-term follow-up of patients who participated in several randomized clinical trials at our center enabled us to perform a detailed retrospective ITT analysis of the long-term outcomes of different immunosuppressive protocols following kidney transplantation.

High rates of deviations from randomized immunosuppression, on the one hand, and the desire to correlate early immunosuppression with late events were the arguments against a per-protocol analysis.

Patients randomized to mTORis after kidney transplantation, either as a replacement for CNIs or in combination with CNIs as a replacement for mycophenolic acid, clearly had inferior tolerability and efficacy, as evidenced by significantly higher discontinuation rates, rejection rates, and the frequent development of dnDSAs. Only a minority of patients were maintained on an originally randomized regimen containing mTORis until graft loss, death, or the end of observation. Patients randomized to receive standard CNI-based immunosuppression experienced lower rejection rates and reduced dnDSA formation, contributing to the overall good tolerability of this regimen.

High dropout rates of patients on mTORis were seen in virtually all trials with this class of immunosuppressants, last reported in the TRANSFORM and Athena studies [13,14,15,16,17]. The adverse effects of sirolimus and everolimus are diverse [46,47] and summarized by Kaplan et al. [48]. Importantly, we observed several serious side-effects, such as 12 pulmonary adverse events (e.g., pneumonitis and interstitial lung disease) as well as acute rejections and the development of dnDSAs, which might have serious consequences for long-term graft survival [3].

Even with a rather short exposure to mTORis, the rate of dnDSA formation significantly increased and was twice as high as that in the comparator arm. In combination with the increased rate of BPARs, this study clearly supports and extends our previous observation [8], which, in 2012, initiated a controversial debate on the efficacy of mTORis after kidney transplantation [49]. One argument against our concerns was the relatively high rate of steroid-free patients at our center, including the speculation that steroid withdrawal in a CNI-free regimen could have contributed to alloimmunization. Our current analysis revealed the opposite effect: steroid withdrawal was associated with a significantly lower rate of dnDSA formation and BPARs. Steroid-free immunosuppression might reflect clinically driven individualization in low-risk patients, but, in combination with better patient and graft survival, it might also reflect an effective reduction in cardiovascular risk and steroid-associated side-effects.

Analyses of graft and patient survival did not support our hypothesis that early randomized immunosuppression significantly determines graft and patient survival. Nevertheless, our data again support the association between dnDSA formation and graft loss (log-rank: *p* < 0.001), and between BPARs and graft loss (log-rank: *p* < 0.001), independent of early immunosuppression. While both mTORi groups had a higher risk of dnDSAs and BPARs, this did not translate into more graft losses. Besides the limited statistical power, a potential reason for this might be that the mTORi treatment was frequently discontinued, and the duration of the initial randomized immunosuppression with mTORis in relation to the total observation time was short. In addition, routine dnDSA screening in our center increased awareness, and, in cases of dnDSA detection, clinicians intervened to prevent ABMR and graft loss [50,51,52].

In part, the unfavorable effects of mTORis on graft survival may also be counterbalanced by CNI nephrotoxicity [53]. However, based on our long-term experience, CNI-free mTORi-based regimens are not a solution for the problem of CNI toxicity. Unfortunately, the combined use of mTORis and CNIs resulted in 30% (23/76 patients) withdrawals due to aggravated nephrotoxicity in the long term. Aggravation of nephrotoxicity with the combination of mTORis and CNIs was observed decades ago [54], and the combined use of tacrolimus and sirolimus [55,56,57] was associated with a high rate of CNI toxicity. Together with mTORi-associated side-effects, this combination had the lowest tolerability and partially explained the high dropout rates of everolimus arms in the TRANSFORM [15] and Athena [17] studies. In summary, the standard CNI regimen with mycophenolic acid clearly provided better tolerability and lower rates of dnDSAs and BPARs.

Recently, five years data from a Brazilian single-center study were published, which suggested a high degree of safety and efficacy in kidney transplant recipients receiving tacrolimus in combination with everolimus [58]. The remarkably low rates of dnDSAs after 5 years in this cohort contrasted with our own data and the results of other groups [59]. An explanation for this surprising observation could be the high rates of zero HLA-DR mismatches (64–76%; the highest rate in the favored rabbit antithymocyte globulin/everolimus/low-tacrolimus/prednisone group), which were, in part, responsible for the good efficacy and low rates of dnDSAs. In our cohort, the rate of zero HLA-DR mismatches was approximately 31%, which was less than half of that in the Brazilian study. There is an increasing body of evidence for the importance of HLA-DR and epitope matching in the development of dnDSAs and rejection [60,61,62].

Not surprisingly, the high recipient age in our cohort was associated with patient survival. Other factors associated with impaired overall patient survival in the multivariate model were non-GN renal disease, male donor sex, and deceased donor type. Importantly, steroid-free immunosuppression reduced the risk of patient death. The detection of dnDSAs per se did not affect patient survival (log-rank: n.s.); however, BPARs significantly decreased overall patient survival (log-rank: *p* = 0.032), regardless of the initial immunosuppressive regimen. The lack of a significant influence of early immunosuppression on overall patient survival contrasts with previous studies from the US registry [11], Australia [12], and a meta-analysis [10], which consistently demonstrated increased mortality in mTORi-treated kidney allograft recipients. In a prospective observational study of 993 prevalent kidney transplant recipients without a history of malignancy, long-term exposure to mTOR inhibitors was associated with significantly increased mortality [9]. Again, patients in our cohort received follow-up in a center with longstanding mTORi experience, with close follow-up and timely interventions in case of efficacy failure and side-effects. This and the impact of competing risks over the long observation period were likely reasons for the lack of a significant influence of early randomized immunosuppression on patient survival.

Subgroups of patients who could potentially benefit from mTORi-based immunosuppression after kidney transplantation have yet to be defined. Univariate analysis of patients from ITT group 2 (mTORi) and ITT group 3 (mTORi + CNI) with above-average duration of randomized immunosuppression in the respective groups revealed that younger, female candidates of a first kidney graft after a longer waiting time might be such a subgroup if they receive an organ from a relatively young donor with a good HLA match (≤three mismatches). Unfortunately, these patients may want to become pregnant and are prone to ovarian cysts; therefore, they are not ideal candidates for mTORi treatment. The incidence of ovarian cysts in our center has been found to be 20.5% and 4.9% in mTORi treatment and control groups, respectively [47].

The present study has some limitations. The patients were recruited from heterogeneous clinical trials at a single center over a 13-year period. Practice patterns have changed with respect to the choice of CNIs, with respect to the target trough levels, and with respect to the monitoring of mycophenolic acid treatment, but the classes of immunosuppressive drugs and their combination have not changed. A significant number of patients did not receive an interleukin-2 receptor induction. Despite the accumulation of data from different immunosuppression trials, the number of mTORi patients was relatively small, especially at the end of the observation period.

Complete long-term follow-up with rather uniform treatment algorithms, the classification of biopsy results according to the most recent Banff classification, and the comparison of randomized and clinically driven mTOR inhibitor use with a CNI-based treatment are the advantages of this study.

From our long-term data, we conclude that the general tolerability and immunosuppressive potential of mTORis in kidney graft recipients is inferior to that of immunosuppression based on the combination of CNIs and mycophenolic acid. The monitoring of mycophenolic acid by measurements of inosine 5′-monophosphate dehydrogenase activity and the definition of individual target CNI trough levels, along with a strict follow-up policy, provide tools for the precise individualization of immunosuppression and good long-term outcomes.

## Figures and Tables

**Table 1:** Clinical immunosuppression trials used to study the long-term effects of early immunosuppression.

Year	Name/ClinicalTrials.gov Identifier/Sponsor	Study Title	Reference	ITT 1 n	ITT 2 n	ITT 3 n
1998	RADB201/Novartis	Parallel-group study of the safety and efficacy of everolimus versus mycophenolate mofetil in de novo renal transplant recipients	[18]	7	-	9
1999	European Tacrolimus/MMF Renal Transplantation Study Group.	Minimization of immunosuppressive therapy after renal transplantation	[19]	12	-	-
2000	NCT00507793/Pfizer	Study Evaluating the Efficacy and Safety of Cyclosporine Reduction in Kidney Transplant Recipients Receiving Sirolimus	[20]	-	-	12
2000	CARMEN Study Group	Corticosteroid-Free Immunosuppression with Tacrolimus, Mycophenolate Mofetil, and Daclizumab Induction	[21]	24	-	-
2001	DACH study/ITT Germany/Austria/Switzerland	Daclizumab, low-dose cyclosporine, mycophenolate mofetil and steroids after kidney transplantation	[22]	14	-	-
2001	NCT00141804/Münster/Astellas	Efficacy and Safety of Sirolimus in Combination with Tacrolimus	-	-	-	4
2002	CRAD001A2307/Novartis	Everolimus with Optimized Cyclosporine Dosing in Renal Transplant Recipients	[18]	-	-	7
2002	myPROMS core D01	Efficacy and Safety of Enteric-Coated Mycophenolate Sodium in De Novo Renal Transplant Recipients	[23]	41	-	-
2002	TERRA Study Group/Astellas	Tacrolimus Combined with Two Different Dosages of Sirolimus in Kidney Transplantation	[24]	5	-	10
2002	NCT00231764/Ekberg, Henrik	MMF, Daclizumab and Corticosteroids as Mainstay Immunosuppression in Renal Transplant Patients	[25]	7	3	-
2003	NCT00239785/Novartis	Efficacy and Safety of FTY720 in de Novo Adult Renal Transplant Recipients	[26]	21	-	-
2003	NCT00189839/Astellas	A Study to Evaluate the Safety and Efficacy of FK506E (MR4) in Patients Undergoing Primary Kidney Transplantation	[27]	11	-	-
2003	NCT00912678/University of Luebeck/Astellas	Minimizing Immunosuppression in Old for Old Kidney Transplantation	[28]	6	-	-
2003	NCT00166244/Erasmus Medical Center/Hoffmann-La Roche	Fixed Dose MMF vs. Concentration Controlled MMF After Renal Transplantation	[29]	54	-	-
2004	NCT00189735/Astellas	A Study to Evaluate FK778 in Kidney Transplant Patients	[30]	4	-	-
2004	NCT00171496/Novartis	Evaluation of Cyclosporine Microemulsion and Tacrolimus on the Rate of New Onset Diabetes Mellitus in Kidney Transplantation Recipients	[31]	9	-	-
2005	NCT00189839/Astellas	A Study to Evaluate the Safety and Efficacy of FK506E (MR4) in Patients Undergoing Primary Kidney Transplantation	[32]	4	-	-
2005	NCT00296309/Astellas	Comparing Efficacy & Safety of Tacrolimus & MMF With/Without Induction in the Elderly Following Kidney Transplantation.	-	14	-	-
2005	NCT00154310/Novartis	Efficacy and Safety of Everolimus with Enteric-Coated Mycophenolate Sodium (EC-MPS) in a Cyclosporine Microemulsionfree Regimen Compared with Standard Therapy in de Novo Renal Transplant Patients	[33]	26	24	-
2006	NCT00256750/Bristol-Myers Squibb	Belatacept Evaluation of Nephroprotection and Efficacy as First-line Immunosuppression (BENEFIT)	[34]	4	-	-
2006	NCT00369278/Novartis	Intensified vs. Standard Dose Therapy with Mycophenolate Sodium Plus Cyclosporin Microemulsion and Corticosteroid Combination in Patients With de Novo Renal Transplant Patients	[35]	53	-	-
2006	NCT00403416/Novartis	Efficacy and Safety of AEB071 Plus Tacrolimus (Converted to Mycophenolic Acid After 3 Months), in Renal Transplant Patients	[36]	8	-	-
2007	NCT00492869/Novartis	Efficacy and Safety of AEB071 versus Tacrolimus in Combination with Mycophenolate Acid Sodium, Basiliximab and Steroids in Preventing Acute Rejection After Kidney Transplantation	[37]	2	-	-
2007	NCT00514514/Novartis	Study Investigating a Standard Regimen in de Novo Kidney Transplant Patients Versus a Calcineurin Inhibitor (CNI)-Free Regimen and a CNI Low Dose Regimen	[38]	39	37	30
2008	NCT00483756/Pfizer	Study of a JAK3 Inhibitor for the Prevention of Acute Rejection in Kidney Transplant Patients	[39]	2	-	-
2009	NCT00956293/Novartis	Study to Evaluate the Efficacy, Safety and Tolerability of Everolimus in de Novo Renal Transplant Recipients Participating in the Eurotransplant Senior Program	[40]	8	17	-
2010	NCT00504543/Novartis	Efficacy, Safety and Tolerability of AEB071 Versus Cyclosporine in the Novo Renal Transplant Recipients	[41]	-	-	4
2010	NCT01064791/Novartis	Efficacy, Safety, Tolerability, and Pharmacokinetics of Sotrastaurin Combined with Tacrolimus vs. a Mycophenolic Acid-tacrolimus Regimen in Renal Transplant Patients	[42]	9	-	-

**Table 2:** Patient baseline characteristics (descriptive data are summarized as median and range).

Characteristic	ITT Group 1: CNI n = 384	ITT Group 2: mTORi n = 81	ITT Group 3: CNI + mTORi n = 76
Age at transplantation (years)	51 (18–74)	54 (20–74)	46 (19–71)
Follow-up (months)	137.3 (3.7–236.4)	111.5 (10.4–185.9)	122.6 (7.1–236.3) *
Recipient gender: male (number; %) female (number; %)	240; 62.5 144; 37.5	38; 46.9 43; 53.1	42; 55.3 34; 44.7 **
Waiting time (months)	46 (0–145)	40 (0–282)	47 (0–144)
Underlying renal disease (GN vs. other)	169 vs. 215	37 vs. 44	39 vs. 37
Number of transplantation: n = 1 (number; %) n > 1 (number; %)	338; 88.0 46; 12.0	75; 92.6 6; 7.4	71; 93.4 5; 6.6
BMI at transplantation (kg/m²)	24.5 (15.1–41.9)	24.5 (16.8–34.6)	23.8 (16.9–39.8)
Donor type: living (number; %) deceased (number; %)	104; 27.1 280; 72.9	20; 24.7 61; 75.3	20; 26.3 56; 73.7
Donor age (years)	53 (18–94)	54 (17–84)	51 (19–74)
Donor gender: male (number; %) female (number; %)	189; 49.2 195; 50.8	35; 43.2 46; 56.8	40; 52.6 36; 47.4
HLA mismatches	3 (0–6)	3 (0–6)	3 (0–6)
Early immunosuppression: tacrolimus (number; %) cyclosporine (number; %) everolimus (number; %) *** sirolimus (number; %) *** mycophenolate (number; %) steroid (number; %)	158; 41.1 226; 58.9 - - 371; 96.6 384; 100	0 78; 96.3 78; 96.3 3; 3.7 81; 100 81; 100	14; 18.4 62; 81.6 50; 65.8 26; 34.2 30; 39.5 **** 76; 100
Steroid withdrawal number; % time after KTX (median; range)	280; 72.9 766; 0–3846	66; 81.5 608; 8–1953	55; 72.4 717; 272–2860 *
Rejections within first month (number; %)	108; 28.1	9; 11.1	18; 23.7 *****

* *p* < 0.01 (median test). ** *p* < 0.05 (Chi-square test). *** mTOR inhibitors were administered at the time of transplantation (n = 49) or replaced CNIs or mycophenolate in the course after transplantation (n = 108). **** mTOR inhibitors replaced mycophenolate initially (n = 46) or after a later switch (n = 30). ***** *p* < 0.01 (Chi-square test).

**Table 3:** Cox proportional hazard modeling of the risks for patient death (a), death-censored graft loss (b), overall graft loss (c), acute rejections (d), and de novo DSA formation (e). Covariates with *p* > 0.20 in the univariate analyses are not reported in the table.

Covariates *	Univariate Analyses			Multivariate Analyses		
	HR	95% CI	*p*	HR	95% CI	*p*
**(a) Patient death**						
Recipient age (≥65 vs. <65)	4.32	3.21–5.82	<0.001	3.48	2.53–4.78	<0.001
Renal disease (GN vs. other)	0.53	0.39–0.72	<0.001	0.54	0.39–0.75	<0.001
Donor age (≥65 vs. <65)	3.13	2.33–4.21	<0.001	-	-	-
Donor gender (female vs. male)	0.82	0.62–1.10	0.185	0.70	0.52–0.95	0.021
Donor type (living vs. deceased)	0.27	0.17–0.44	<0.001	0.47	0.29–0.76	0.002
Number of mismatches (4–6 vs. 0–3)	2.06	1.54–2.76	<0.001	-	-	-
KTX function (DGF vs. primary function)	1.88	1.40–2.53	<0.001	-	-	-
ITT group (mTORi vs. CNI; CNI + mTORi vs. CNI)	0.74 0.97	0.45–1.23 0.64–1.47	0.252 0.891	-	-	-
Steroid stop (yes vs. no)	0.34	0.25–0.46	<0.001	0.32	0.24–0.44	<0.001
**(b) Death-censored graft loss**						
Recipient age (≥65 vs. <65)	2.52	1.71–3.70	<0.001	2.59	1.73–3.88	<0.001
Number of KTXs (repeated vs. first)	2.03	1.29–3.19	0.002	1.82	1.14–2.91	0.012
Pretransplant PRAs (positive vs. negative)	1.93	1.04–3.58	0.038	-	-	-
Donor age (≥65 vs. <65)	2.26	1.55–3.29	<0.001	-	-	-
Donor type (living vs. deceased)	0.39	0.24–0.63	<0.001	-	-	-
Number of mismatches (4–6 vs. 0–3)	1.97	1.37–2.82	<0.001	-	-	-
KTX function (DGF vs. primary function)	2.38	1.67–3.41	<0.001	1.90	1.32–2.73	<0.001
BPAR within first month (yes vs. no)	1.73	1.20–2.49	0.003	-	-	-
ITT group (mTORi vs. CNI; CNI + mTORi vs. CNI)	1.20 1.07	0.72–1.99 0.64–1.80	0.493 0.799	- -	- -	- -
Steroid stop (yes vs. no)	0.27	0.19–0.38	<0.001	0.28	0.19–0.40	<0.001
**(c) Overall graft loss**						
Recipient age (≥65 vs. <65)	3.17	2.43–4.15	<0.001	2.77	2.07–3.70	<0.001
Number of KTXs (repeated vs. first)	1.43	0.99–2.06	0.056	-	-	-
Pretransplant PRAs (positive vs. negative)	1.56	0.96–2.53	0.069	-	-	-
Donor age (≥65 vs. <65)	2.51	1.92–3.28	<0.001	-	-	-
Donor type (living vs. deceased)	0.34	0.24–0.49	<0.001	0.56	0.38–0.83	0.004
Number of mismatches (4–6 vs. 0–3)	1.85	1.43–2.06	<0.001	-	-	-
KTX function (DGF vs. primary function)	2.03	1.56–2.63	<0.001	1.47	1.12–1.93	0.006
BPAR within first month (yes vs. no)	1.39	1.06–1.83	0.017	-	-	-
ITT group (mTORi vs. CNI; CNI + mTORi vs. CNI)	0.99 1.09	0.67–1.47 0.76–1.58	0.955 0.634	- -	- -	- -
Steroid stop (yes vs. no)	0.27	0.21–0.35	<0.001	0.26	0.20–0.34	<0.001
Renal disease (GN vs. other)	0.68	0.52–0.88	0.003	0.63	0.48–0.83	0.001
KTX time point (after vs. before 31 March 2005)	0.83	0.64–1.08	0.17	0.74	0.57–0.97	0.03
**(d) BPAR episodes after first month**						
Pretransplant PRAs (positive vs. negative)	1.54	0.81–2.93	0.188	-	-	-
Number of mismatches (4–6 vs. 0–3)	1.61	1.15–2.24	0.005	1.53	1.10–2.14	0.013
KTX function (DGF vs. primary function)	1.54	1.09–2.16	0.013	1.46	1.04–2.06	0.029
BPAR within first month (yes vs. no)	1.50	1.06–2.12	0.023	-	-	-
ITT group (mTORi vs. CNI; CNI + mTORi vs. CNI)	1.81 2.12	1.19–2.76 1.41–3.20	0.006 <0.001	2.02 2.21	1.32–3.09 1.47–3.34	0.001 <0.001
Steroid stop (yes vs. no)	0.35	0.25–0.49	<0.001	0.36	0.26–0.50	<0.001
**(e) De novo DSA formation**						
Recipient age (≥65 vs. <65)	1.50	1.03–2.19	0.037	-	-	-
Number of KTXs (repeated vs. first)	1.59	1.01–2.49	0.045	-	-	-
Donor age (≥65 vs. <65)	1.40	0.98–2.02	0.068	-	-	-
Number of mismatches (4–6 vs. 0–3)	2.61	1.90–3.58	<0.001	2.48	1.80–3.43	<0.001
KTX function (DGF vs. primary function)	1.61	1.15–2.26	0.005	1.45	1.03–2.03	0.030
BPAR within first month (yes vs. no)	1.26	0.89–1.78	0.195	-	-	-
ITT group (mTORi vs CNI; CNI + mTORi vs. CNI)	1.75 1.94	1.16–2.64 1.28–2.94	0.008 0.002	1.59 2.10	1.02–2.48 1.38–3.19	0.042 <0.001
Steroid stop (yes vs. no)	0.47	0.33–0.67	<0.001	0.47	0.33–0.67	<0.001
KTX time point (after vs. before March 31th 2005)	1.73	1.24–2.41	0.001	1.49	1.03–2.15	0.033

* The last category in parentheses is the reference category. BPAR: biopsy-proven acute rejection; CI: confidence interval; CNI: calcineurin inhibitor; DGF: delayed graft function; DSAs: donor-specific HLA antibodies; GN: glomerulonephritis; HR: hazard ratio; ITT: intention-to-treat; KTX: kidney transplantation; mTORi: mammalian target of rapamycin inhibitor(s); PRAs: panel-reactive antibodies.

## Data Availability

The raw data supporting the conclusions of this article will be made available by the authors on request.

## List of Abbreviations

ABMR	antibody-mediated rejection
BPAR	biopsy-proven acute rejection
CI	confidence interval
CNI	calcineurin inhibitor
dnDSAs	de novo donor-specific HLA antibodies
GN	glomerulonephritis
HLA	human leukocyte antigen(s)
HR	hazard ratio
ITT	intention to treat
KTX	kidney transplantation
MMF	mycophenolate mofetil
MPA	mycophenolic acid
mTORi	mammalian target of rapamycin inhibitor(s)
n.s.	not significant
PRAs	panel-reactive antibodies

## References

[1] Azzi J.R., Sayegh M.H., Mallat S.G. (2013). Calcineurin Inhibitors: 40 Years Later, Can’t Live without…. J. Immunol..

[2] Eckardt K.U., Kasiske B.L. (2009). Kidney disease: Improving global outcomes. Am. J. Transplant..

[3] Mayrdorfer M., Liefeldt L., Wu K., Rudolph B., Zhang Q., Friedersdorff F., Lachmann N., Schmidt D., Osmanodja B., Naik M.G. (2021). Exploring the Complexity of Death-Censored Kidney Allograft Failure. J. Am. Soc. Nephrol..

[4] Sehgal S.N., Baker H., Vézina C. (1975). Rapamycin (AY-22,989), a new antifungal antibiotic. II. Fermentation, isolation and characterization. J. Antibiot..

[5] Kahan B.D. (2000). Efficacy of sirolimus compared with azathioprine for reduction of acute renal allograft rejection: A randomised multicentre study. The Rapamune US Study Group. Lancet.

[6] (2019). Product Information: Rapamune (sirolimus) Oral Solution and Tablets.

[7] (2021). Product Information: Zortress (everolimus) Tablets.

[8] Liefeldt L., Brakemeier S., Glander P., Waiser J., Lachmann N., Schönemann C., Zukunft B., Illigens P., Schmidt D., Wu K. (2012). Donor-specific HLA antibodies in a cohort comparing everolimus with cyclosporine after kidney transplantation. Am. J. Transplant..

[9] Cortazar F., Molnar M.Z., Isakova T., Czira M.E., Kovesdy C.P., Roth D., Mucsi I., Wolf M. (2012). Clinical outcomes in kidney transplant recipients receiving long-term therapy with inhibitors of the mammalian target of rapamycin. Am. J. Transplant..

[10] Knoll G.A., Kokolo M.B., Mallick R., Beck A., Buenaventura C.D., Ducharme R., Barsoum R., Bernasconi C., Blydt-Hansen T.D., Ekberg H. (2014). Effect of sirolimus on malignancy and survival after kidney transplantation: Systematic review and meta-analysis of individual patient data. BMJ.

[11] Isakova T., Xie H., Messinger S., Cortazar F., Scialla J.J., Guerra G., Contreras G., Roth D., Burke G.W., Molnar M.Z. (2013). Inhibitors of mTOR and risks of allograft failure and mortality in kidney transplantation. Am. J. Transplant..

[12] Badve S.V., Pascoe E.M., Burke M., Clayton P.A., Campbell S.B., Hawley C.M., Lim W.H., McDonald S.P., Wong G., Johnson D.W. (2016). Mammalian Target of Rapamycin Inhibitors and Clinical Outcomes in Adult Kidney Transplant Recipients. Clin. J. Am. Soc. Nephrol..

[13] Pascual J., Berger S.P., Witzke O., Tedesco H., Mulgaonkar S., Qazi Y., Chadban S., Oppenheimer F., Sommerer C., Oberbauer R. (2018). Everolimus with Reduced Calcineurin Inhibitor Exposure in Renal Transplantation. J. Am. Soc. Nephrol..

[14] Tedesco-Silva H., Pascual J., Viklicky O., Basic-Jukic N., Cassuto E., Kim D.Y., Cruzado J.M., Sommerer C., Bakr M.A., Garcia V.D. (2019). Safety of Everolimus With Reduced Calcineurin Inhibitor Exposure in De Novo Kidney Transplants: An Analysis From the Randomized TRANSFORM Study. Transplantation.

[15] Berger S.P., Sommerer C., Witzke O., Tedesco H., Chadban S., Mulgaonkar S., Qazi Y., de Fijter J.W., Oppenheimer F., Cruzado J.M. (2019). Two-year outcomes in de novo renal transplant recipients receiving everolimus-facilitated calcineurin inhibitor reduction regimen from TRANSFORM study. Am. J. Transplant..

[16] Chadban S., Tedesco-Silva H. (2019). ATHENA: Wisdom and warfare in defining the role of de novo mTOR inhibition in kidney transplantation. Kidney Int..

[17] Sommerer C., Suwelack B., Dragun D., Schenker P., Hauser I.A., Witzke O., Hugo C., Kamar N., Merville P., Junge M. (2019). An open-label, randomized trial indicates that everolimus with tacrolimus or cyclosporine is comparable to standard immunosuppression in de novo kidney transplant patients. Kidney Int..

[18] Vitko S., Tedesco H., Eris J., Pascual J., Whelchel J., Magee J.C., Campbell S., Civati G., Bourbigot B., Alves Filho G. (2004). Everolimus with Optimized Cyclosporine Dosing in Renal Transplant Recipients: 6-Month Safety and Efficacy Results of Two Randomized Studies. Am. J. Transplant..

[19] Vanrenterghem Y., van Hooff J.P., Squifflet J.P., Salmela K., Rigotti P., Jindal R.M., Pascual J., Ekberg H., Sicilia L.S., Boletis J.N. (2005). Minimization of immunosuppressive therapy after renal transplantation: Results of a randomized controlled trial. Am. J. Transplant..

[20] Mühlbacher F., Neumayer H.H., del Castillo D., Stefoni S., Zygmunt A.J., Budde K., European Rapamune Cyclosporine Minimization Study Group (2014). The efficacy and safety of cyclosporine reduction in de novo renal allograft patients receiving sirolimus and corticosteroids: Results from an open-label comparative study. Transpl. Int..

[21] Rostaing L., Cantarovich D., Mourad G., Budde K., Rigotti P., Mariat C., Margreiter R., Capdevilla L., Lang P., Vialtel P. (2005). CARMEN Study Group. Corticosteroid-free immunosuppression with tacrolimus, mycophenolate mofetil, and daclizumab induction in renal transplantation. Transplantation.

[22] Fangmann J., Arns W., Marti H.P., Hauss J., Ketteler M., Beckurts T., Boesmueller C., Pohanka E., Martin P.Y., Gerhardt M. (2010). Impact of daclizumab, low-dose cyclosporine, mycophenolate mofetil and steroids on renal function after kidney transplantation. Nephrol. Dial. Transplant..

[23] Einecke G., Schütz M., Mai I., Fritsche L., Giessing M., Glander P., Neumayer H.H., Budde K. (2005). Limitations of C2 monitoring in renal transplant recipients. Nephrol. Dial. Transplant..

[24] Vitko S., Wlodarczyk Z., Kyllönen L., Czajkowski Z., Margreiter R., Backman L., Perner F., Rigotti P., Jaques B., Abramowicz D. (2006). Tacrolimus Combined with Two Different Dosages of Sirolimus in Kidney Transplantation: Results of a Multicenter Study. Am. J. Transplant..

[25] Ekberg H., Tedesco-Silva H., Demirbas A., Vítko S., Nashan B., Gürkan A., Margreiter R., Hugo C., Grinyó J.M., Frei U. (2007). Reduced exposure to calcineurin inhibitors in renal transplantation. N. Engl. J. Med..

[26] Salvadori M., Budde K., Charpentier B., Klempnauer J., Nashan B., Pallardo L.M., Eris J., Schena F.P., Eisenberger U., Rostaing L. (2006). FTY720 0124 Study Group. FTY720 versus MMF with cyclosporine in de novo renal transplantation: A 1-year, randomized controlled trial in Europe and Australasia. Am. J. Transplant..

[27] Krämer B.K., Albano L., Banas B., Charpentier B., Bäckman L., Tedesco-Silva H., Lehner F., Mondragón-Ramírez G.A., Glyda M., Cassuto-Viguier E. (2017). Efficacy of Prolonged- and Immediate-release Tacrolimus in Kidney Transplantation: A Pooled Analysis of Two Large, Randomized, Controlled Trials. Transplant. Proc..

[28] Meier M., Bode W., Nitschke M., Wong W., Ison M.G., Kramer J., Jabs W., Bürk C.G., Lehnert H. (2011). High Rejection Rates with Low Dose Immunosuppression in Old for Old Kidney Transplantation. Transplantationsmedizin.

[29] Van Gelder T., Silva H.T., de Fijter J.W., Budde K., Kuypers D., Tyden G., Lohmus A., Sommerer C., Hartmann A., Le Meur Y. (2008). Comparing mycophenolate mofetil regimens for de novo renal transplant recipients: The fixed-dose concentration-controlled trial. Transplantation.

[30] Wlodarczyk Z., Vanrenterghem Y., Krämer B.K., Squifflet J.P., Ostrowski M. (2012). A multicenter, randomized, double-blind study comparing different FK778 doses (manitimus) with tacrolimus and steroids vs. MMF with tacrolimus and steroids in renal transplantation. BMC Nephrol..

[31] Vincenti F., Friman S., Scheuermann E., Rostaing L., Jenssen T., Campistol J.M., Uchida K., Pescovitz M.D., Marchetti P., Tuncer M. (2007). Results of an international, randomized trial comparing glucose metabolism disorders and outcome with cyclosporine versus tacrolimus. Am. J. Transplant..

[32] Krämer B.K., Charpentier B., Bäckman L., Silva H.T., Mondragon-Ramirez G., Cassuto-Viguier E., Mourad G., Sola R., Rigotti P., Mirete J.O. (2010). Tacrolimus once daily (ADVAGRAF) versus twice daily (PROGRAF) in de novo renal transplantation: A randomized phase III study. Am. J. Transplant..

[33] Budde K., Becker T., Arns W., Sommerer C., Reinke P., Eisenberger U., Kramer S., Fischer W., Gschaidmeier H., Pietruck F. (2011). Everolimus-based, calcineurin-inhibitor-free regimen in recipients of de-novo kidney transplants: An open-label, randomised, controlled trial. Lancet.

[34] Vincenti F., Larsen C.P., Alberu J., Bresnahan B., Garcia V.D., Kothari J., Lang P., Urrea E.M., Massari P., Mondragon-Ramirez G. (2012). Three-year outcomes from BENEFIT, a randomized, active-controlled, parallel-group study in adult kidney transplant recipients. Am. J. Transplant..

[35] Glander P., Sommerer C., Arns W., Ariatabar T., Kramer S., Vogel E.M., Shipkova M., Fischer W., Zeier M., Budde K. (2010). Pharmacokinetics and pharmacodynamics of intensified versus standard dosing of mycophenolate sodium in renal transplant patients. Clin. J. Am. Soc. Nephrol..

[36] Budde K., Sommerer C., Becker T., Asderakis A., Pietruck F., Grinyo J.M., Rigotti P., Dantal J., Ng J., Barten M.J. (2010). Sotrastaurin, a Novel Small Molecule Inhibiting Protein Kinase C: First Clinical Results in Renal-Transplant Recipients. Am. J. Transplant..

[37] Friman S., Arns W., Nashan B., Vincenti F., Banas B., Budde K., Cibrik D., Chan L., Klempnauer J., Mulgaonkar S. (2011). Sotrastaurin, a novel small molecule inhibiting protein-kinase C: Randomized phase II study in renal transplant recipients. Am. J. Transplant..

[38] Budde K., Zeier M., Witzke O., Arns W., Lehner F., Guba M., Jacobi J., Kliem V., Reinke P., Hauser I.A. (2017). Everolimus with cyclosporine withdrawal or low-exposure cyclosporine in kidney transplantation from Month 3: A multicentre, randomized trial. Nephrol. Dial. Transplant..

[39] Vincenti F., Tedesco-Silva H., Busque S., O’Connell P., Friedewald J., Cibrik D., Budde K., Yoshida A., Cohney S., Weimar W. (2012). Randomized Phase 2b Trial of Tofacitinib (CP-690,550) in De Novo Kidney Transplant Patients: Efficacy, Renal Function and Safety at 1 Year. Am. J. Transplant..

[40] Brakemeier S., Arns W., Lehner W., Witzke O., Vonend O., Sommerer C., Mühlfeld A., Rath T., Schuhmann R., Zukunft B. (2019). Everolimus in de novo kidney transplant recipients participating in the Eurotransplant senior program: Results of a prospective randomized multicenter study (SENATOR). PLoS ONE.

[41] Tedesco-Silva H., Kho M.M., Hartmann A., Vitko S., Russ G., Rostaing L., Budde K., Campistol J.M., Eris J., Krishnan I. (2013). Sotrastaurin in calcineurin inhibitor-free regimen using everolimus in de novo kidney transplant recipients. Am. J. Transplant..

[42] Russ G.R., Tedesco-Silva H., Kuypers D.R., Cohney S., Langer R.M., Witzke O., Eris J., Sommerer C., von Zur-Mühlen B., Woodle E.S. (2013). Efficacy of sotrastaurin plus tacrolimus after de novo kidney transplantation: Randomized, phase II trial results. Am. J. Transplant..

[43] Sis B., Mengel M., Haas M., Colvin R.B., Halloran P.F., Racusen L.C., Solez K., Baldwin W.M., Bracamonte E.R., Broecker V. (2010). Banff ’09 meeting report: Antibody mediated graft deterioration and implementation of Banff working groups. Am. J. Transplant..

[44] Haas M., Loupy A., Lefaucheur C., Roufosse C., Glotz D., Seron D., Nankivell B.J., Halloran P.F., Colvin R.B., Akalin E. (2018). The Banff 2017 kidney meeting report: Revised diagnostic criteria for chronic active T-cell-mediated rejection, antibody-mediated rejection, and prospects for integrative endpoints for next-generation clinical trials. Am. J. Transplant..

[45] Schmidt D., Osmanodja B., Pfefferkorn M., Graf V., Raschke D., Duettmann W., Naik M.G., Gethmann C.J., Mayrdorfer M., Halleck F. (2021). TBase—An Integrated Electronic Health Record and Research Database for Kidney Transplant Recipients. J. Vis. Exp..

[46] Hamdy A.F., Bakr M.A., Ghoneim M.A. (2010). Proteinuria among primarily sirolimus treated live-donor renal transplant recipients’ long-term experience. Exp. Clin. Transplant..

[47] Bachmann F., Glander P., Budde K., Bachmann C. (2018). High Incidence of Ovarian Cysts in Women Receiving mTOR Inhibitors After Renal Transplantation. J. Womens Health.

[48] Kaplan B., Qazi Y., Wellen J.R. (2014). Strategies for the management of adverse events associated with mTOR inhibitors. Transplant. Rev..

[49] Pascual J., Arns W. (2012). Does everolimus increase donor-specific HLA antibodies in kidney transplant recipients?. Am. J. Transplant..

[50] Waiser J., Duerr M., Budde K., Rudolph B., Wu K., Bachmann F., Halleck F., Schönemann C., Lachmann N. (2017). Treatment of Acute Antibody-Mediated Renal Allograft Rejection With Cyclophosphamide. Transplantation.

[51] Waiser J., Klotsche J., Lachmann N., Wu K., Rudolph B., Halleck F., Liefeldt L., Bachmann F., Budde K., Duerr M. (2020). Predictors of graft survival at diagnosis of antibody-mediated renal allograft rejection: A retrospective single-center cohort study. Transpl. Int..

[52] Doberer K., Duerr M., Halloran P.F., Eskandary F., Budde K., Regele H., Reeve J., Borski A., Kozakowski N., Reindl-Schwaighofer R. (2021). A Randomized Clinical Trial of Anti-IL-6 Antibody Clazakizumab in Late Antibody-Mediated Kidney Transplant Rejection. J. Am. Soc. Nephrol..

[53] Gallagher M., Jardine M., Perkovic V., Cass A., McDonald S., Petrie J., Eris J. (2009). Cyclosporine withdrawal improves long-term graft survival in renal transplantation. Transplantation.

[54] Vítko S., Margreiter R., Weimar W., Dantal J., Kuypers D., Winkler M., Øyen O., Viljoen H.G., Filiptsev P., Sadek S. (2005). Three-year efficacy and safety results from a study of everolimus versus mycophenolate mofetil in de novo renal transplant patients. Am. J. Transplant..

[55] Hamdy. A.F., El-Agroudy A.E., Bakr M.A., Mostafa A., El-Baz M., El-Shahawy E.M., Ghoneim M.A. (2005). Comparison of sirolimus with low-dose tacrolimus versus sirolimus-based calcineurin inhibitor-free regimen in live donor renal transplantation. Am. J. Transplant..

[56] Hamdy A.F., Bakr M.A., Ghoneim M.A. (2008). Long-term efficacy and safety of a calcineurin inhibitor-free regimen in live-donor renal transplant recipients. J. Am. Soc. Nephrol..

[57] Hamdy A.F., Elhadedy M.A., Donia A.F., Taha N.M., Bakr M.A. (2019). Outcome of sirolimus-based immunosuppression; fifteen years post-live-donor kidney transplantation: Single-center experience. Clin. Transplant..

[58] Ficher K.N., Dreige Y., Gessolo Lins P.R., Ferreira A.N., de Rezende Freschi J.T., Linhares K., Martins S.S., Custodio L., Cristelli M., Viana L. (2022). Long-term Efficacy and Safety of Everolimus Versus Mycophenolate in Kidney Transplant Recipients Receiving Tacrolimus. Transplantation.

[59] Wiebe C., Gibson I.W., Blydt-Hansen T.D., Pochinco D., Birk P.E., Ho J., Karpinski M., Goldberg A., Storsley L., Rush D.N. (2015). Rates and determinants of progression to graft failure in kidney allograft recipients with de novo donor-specific antibody. Am. J. Transplant..

[60] Wiebe C., Pochinco D., Blydt-Hansen T.D., Ho J., Birk P.E., Karpinski M., Goldberg A., Storsley L.J., Gibson I.W., Rush D.N. (2013). Class II HLA epitope matching-A strategy to minimize de novo donor-specific antibody development and improve outcomes. Am. J. Transplant..

[61] Hricik D.E., Formica R.N., Nickerson P., Rush D., Fairchild R.L., Poggio E.D., Gibson I.W., Wiebe C., Tinckam K., Bunnapradist S. (2015). Adverse Outcomes of Tacrolimus Withdrawal in Immune-Quiescent Kidney Transplant Recipients. J. Am. Soc. Nephrol..

[62] Wiebe C., Kosmoliaptsis V., Pochinco D., Gibson I.W., Ho J., Birk P.E., Goldberg A., Karpinski M., Shaw J., Rush D.N. (2019). HLA-DR/DQ molecular mismatch: A prognostic biomarker for primary alloimmunity. Am. J. Transplant..

